# PD-1 inhibitor-associated type 1 diabetes: A case report and systematic review

**DOI:** 10.3389/fpubh.2022.885001

**Published:** 2022-08-05

**Authors:** Cuiping Lin, Xuan Li, Yu Qiu, Zheng Chen, Jianping Liu

**Affiliations:** Department of Endocrinology and Metabolism, Second Affiliated Hospital of Nanchang University, Nanchang, China

**Keywords:** PD-1 inhibitors, diabetes, immune checkpoint inhibitor, camrelizumab, insulin

## Abstract

**Objective:**

This study aimed to summarize the clinical characteristics of programmed death receptor 1 (PD-1) inhibitor-associated type 1 diabetes so as to improve the ability of clinicians to correctly diagnose and treat it.

**Methods:**

We reported a case of a 70-year-old woman with gastric cancer who developed hyperosmolar hyperglycemic coma during camrelizumab (a PD-1 inhibitor) treatment and was diagnosed with PD-1 inhibitor-associated type 1 diabetes. We conducted a systematic review of 74 case reports of type 1 diabetes associated with PD-1 inhibitor therapy published before June 2022.

**Results:**

The patient developed type 1 diabetes with hyperosmolar hyperglycemic coma after receiving camrelizumab chemotherapy for 6 months (9 cycles). We searched 69 English articles comprising 75 patients, all of whom had been treated with a PD-1 inhibitor (nivolumab or pembrolizumab) and progressed to diabetes after an average of 6.11 (1–28) cycles. Nivolumab combined with ipilimumab (a cytotoxic T lymphocyte-associated protein 4 inhibitor) had the shortest onset (4.47 cycles on average). A total of 76% (57/75) of patients developed diabetic ketoacidosis (DKA) at onset, and 50.67% (38/75) of patients had C-peptide <0.1 ng/mL. Most of the patients were tested for insulin autoantibodies, with a positive rate of 33.33% (23/69); of these, 86.96% (20/23) were tested for glutamate decarboxylase antibody and 46.67% (35/75) were tested for human leukocyte antigen (HLA). HLA-DR4 was the most common type.

**Conclusions:**

The progression of type 1 diabetes induced by PD-1 inhibitors is relatively rapid. Islet failure often occurs when detected, seriously endangering patients' lives. Patients treated with PD-1 inhibitors should closely monitor their plasma glucose level during treatment to detect, diagnose, and treat diabetes on time.

## Introduction

Immune checkpoint inhibitors play an important role in immune tolerance through negative regulation of the immune system. Common immune checkpoints include programmed death receptor 1 (PD-1) and cytotoxic T lymphocyte-associated protein 4 (CTLA-4). PD-1 belongs to the B7-CD28 family in the –immunoglobulin superfamily. However, PD-1 does not share ligands with CD28 (1). The specific ligands of PD-1 include PD-L1 (B7-H1) and PD-L2 (B7-DC). The binding of PD-1 and its ligand can prevent T-cell proliferation, cytokine production, and cell decomposition. Camrelizumab is a novel immune checkpoint inhibitor (PD-1 inhibitor) with independent intellectual property rights in China. A highly humanized monoclonal antibody, IgG4 is widely used in tumor treatment. Its pharmacological action is to block the PD-1/PD-L1 pathway by binding to PD-1 and hence promote T-cell activation and proliferation, thereby inhibiting tumor growth (2). A PD-1 inhibitor overactivated the immune system and its ligand is widely expressed in hematopoietic cells, pancreatic cells, macrophages, and dendritic cells (3, 4). Therefore, may affect other tissue cells during treatment. A series of immune-related adverse events (irAEs) are induced, the most common of which are pituitary and thyroid dysfunction, diabetes, and adrenal hypofunction (5). In type 1 diabetes (T1DM), an autoimmune disease, the destruction of islet β cells leads to absolute insulin deficiency (6). Type 1 diabetes, a rare irAE associated with PD-1 inhibitors, has an estimated incidence of 0.2–1.4% (7–9). PD-1 inhibitors indirectly damage many islet β-cells after the overactivation of the autoimmune system, resulting in the development of diabetes. We reported a case of a 70-year-old woman with gastric cancer who developed type 1 diabetes complicated by hyperosmolar hyperglycemic coma 6 months after receiving camrelizumab chemotherapy. Subsequently, we conducted a systematic review and summary of published case reports to draw the attention of clinicians to this disease and thus its diagnosis and treatment.

## Case presentation

A 70-year-old female patient underwent a total gastrectomy for gastric cancer in 2017. Multiple lymph node metastases of gastric cancer were found in August 2019. Since April 2020, she has regularly received 200 mg camrelizumab intravenously, 21 days/cycle, combined with apatinib mesylate tablets 850 mg/day at a local cancer hospital. No obvious adverse reactions were noted, except pancytopenia, during the treatment. On the night of October 12, 2020, the patient drank many sugary drinks. The next morning, her family found her unconscious and took her to a local hospital. The auxiliary examination revealed the following: random plasma glucose was 1,082 mg/dL, HbA1c was 7.88%, fasting C-peptide was 0.04 ng/mL, blood sodium was 140.6 mmol/L, blood potassium was 6.46 mmol/L, blood chlorine was 97.4 mmol/L, effective plasma osmotic pressure was 354.22 mOsm/L (>320 mOsm/L), urine glucose was (3+), and urine ketone body was (+-); arterial blood gas: pH 7.44, bicarbonate (${\text{HCO}}_{3}^{-}$) 19.7 mmol/ L, and base excess −3.3 mmol/L. The diagnosis included the following: (1) hyperosmolar hyperglycemic coma; (2) hyperkalemia; (3) diabetes; (4) metabolic acidosis. The patient was treated for fluid replenishment, hypoglycemia, and correction of water and electrolyte disorders. After discharge, the patient received insulin glargine 8U to lower hyperglycemia, but the patient had poor plasma glucose control. On October 26, 2020, she was admitted to our outpatient department for further diagnosis and treatment. The physical examination showed the following: body temperature 36.2°C, pulse 64 beats/min, 20 breaths/min, blood pressure 110/62 mm Hg (1 mm Hg = 0.133 kPa), height 150 cm, body weight 41 kg, and body mass index (BMI) 18.22 kg/m^2^. The examination of the heart, lung, and abdomen revealed no abnormality, with no edema in both lower extremities. The family denied a history of diabetes. During admission, the fasting plasma glucose was 440 mg/dL, 1-h postprandial plasma glucose level was 239 mg/dL, 2-h postprandial plasma glucose level was 212 mg/dL, HbA1c was 11.1%, IA-2A was positive, serum C-peptide (0, 60, and 120 min) was <0.1 ng/mL, and urinary ketone body was (–) (Tables 1, 2). Combined with the fact that no report on diabetes caused by apatinib mesylate tablets was shown, this patient was diagnosed with PD-1 inhibitor-associated type 1 diabetes. The patient's islet function was poor, and the presentation was marked by brittle diabetes. Therefore, intensive treatment with an insulin pump was given after admission: 10 units of basal insulin and 4 units of insulin aspart before breakfast, lunch, and dinner. The patient's plasma glucose level was continuously monitored, and the insulin dose was adjusted according to the plasma glucose level. After the plasma glucose level was stable, the patient was treated with low-dose rapid-acting insulin before three meals and long-acting insulin before sleep, including 6 units of insulin aspart before breakfast, 5 units before lunch, and 5 units before dinner, together with 6 units of insulin degludec before bed.

**Table 1:** Laboratory parameters at the time of hospital admission.

**Laboratory Test**	**Value**	**Reference range**
Plasma glucose (mg/dL)	440	70–110
HbA1c (%)	11.1	3–6
Na^+^ (mmol/L)	131.72	137–147
K^+^ (mmol/L)	4.49	3.5–5.3
Cl^+^ (mmol/L)	103.62	99–110
CO_2_CP (mmol/L)	21.42	23–29
WBC (109^/L)	1.99	3.5–9.5
RBC (101^2/L)	2.59	3.8–5.1
Hb (g/L)	88	110–150
PLT (109^/L)	112	125–350
Urinary glucose	2+	-
Urinary ketone	-	-
Urinary pH	6.0	4.5–8.0
Urinary ACR (mmol/L)	9.25	<30

*HbA1c, Glycated hemoglobin; CO_2_CP, CO_2_ combining power; WBC, white blood cell; RBC, red blood cell; Hb, hemoglobin; PLT, platelets; ACR, albumin–creatinine ratio*.

**Table 2:** Related autoantibodies and islet function.

**Laboratory test**	**Value**	**Reference range**
GADA	-	-
ICA	-	-
IA-2A	+	-
IAA	-	-
ZnT8A	-	-
Plasma glucose 0 min (mg/dL)	440	70–110
Plasma glucose 60 min (mg/dL)	239	79.2–140.4
Plasma glucose 120 min (mg/dL)	212	79.2–140.4
Serum C peptide 0 min (ng/mL)	0.06	1.1–4.4
Serum C peptide 60 min (ng/mL)	0.04	
Serum C peptide 120 min (ng/mL)	0.07	

*GADA, Glutamic acid decarboxylase antibody; ICA, islet-cell antibodies; IA-2A, insulinoma-associated antigen-2; IAA, insulin autoantibody; ZnT8A, zinc transporter 8 antibody*.

## Methods

We searched English-language case reports on PD-1 inhibitors and diabetes published before June 2022, using the search terms “PD-1 inhibitor”, “Nivolumab”, “Pembrolizumab”, or “Immune checkpoint inhibitor” and “Diabetes”, “Diabetes Mellitus”, “diabetic ketoacidosis”, “ketoacidosis” and “DKA”. A total of 69 reports (10–78), were retrieved. The following information was extracted from each case: author, year of publication, patient's age, gender, tumor type, type of immune checkpoint inhibitor, onset cycle, plasma glucose level, HbA1c, C-peptide, presence of DKA, islet autoantibodies, and human leukocyte antigen (HLA) genotypes (Supplementary Table S1). The informed consent of the patient herself has been obtained for this case report.

## Results

We searched 69 English articles, comprising 75 patients. All patients received PD-1 inhibitors therapy, with a female/male ratio of 23/52 and an average age of 63 (12–85) years (Supplementary Table S2). Tumor types included melanoma 36% (27/75), non-small-cell lung cancer 16% (12/75), renal cell carcinoma 12% (9/60), and other types 36% (27/75) (Supplementary Table S2). It was preferable to calculate by cycle due to the inconsistent duration of drug use, with an average of 6.11 (1–28) cycles for the diagnosis of diabetes (Supplementary Table S2). The mean plasma glucose level was 656 (271–1,298) mg/dL, and the mean HbA1c was 7.85% (6.1%−11.1%) (Supplementary Table S2). A majority of patients, 76% (57/75), had DKA at the onset. The reference range of C-peptide detection was inconsistent in most patients; except for some for whom the value was not explained, 59.38% (38/64) of patients had C-peptide <0.1 ng/mL or undetectable (Supplementary Table S2). A total of 93.33% (70/75) of patients were tested for insulin autoantibodies, with a positive rate of 32.86% (23/70) (Supplementary Table S2). The average progression of diabetes was 3.39 cycles in patients who were antibody positive and 7.5 cycles in patients who were antibody negative. Glutamate decarboxylase antibody (GADA) accounted for 86.96% (20/23) of the autoantibodies (Supplementary Table S2). Two or more antibodies were positive in 30.43% (7/23) of patients. About 46.67% (35/75) of the patients were tested for HLA genotypes, mainly HLA-DR4 (37.14%) (Supplementary Tables S1, S2). HLA gene testing was not performed in the present case. Also, further analysis of the use of immune checkpoint inhibitors suggested that patients receiving nivolumab developed diabetes in an average of 6.47 cycles, whereas those receiving pembrolizumab developed diabetes in a longer duration (6.5 cycles). Nivolumab also resulted in lower mean plasma glucose level and HbA1c than pembrolizumab. Patients treated with the PD-1 inhibitor nivolumab in combination with the CTLA-4 inhibitor ipilimumab had the shortest time to diagnose of diabetes (4.47 cycles on average) (Supplementary Table S3). Among the cases we collected, two patients received PD-1 inhibitor treatment and were diagnosed with diabetes after a period of drug withdrawal (61, 73). The guidelines promulgated by the European Society for Medical Oncology note that another plasma glucose measurement 4–6 weeks after the last cycle of immunotherapy may be necessary (79).

## Discussion

The irAEs induced by ICPIs mainly include thyroid dysfunction, hypophysitis, adrenal hypofunction, diabetes, etc., (5). Multiple endocrinopathies induced by PD-1 inhibitors can possibly occur at the same time, despite the low frequency of adverse events in each endocrine organ. Our results showed that 41.33% (31/75) patients were complicated with other endocrine gland abnormalities, among which thyroid dysfunction (34.67%) was the most common, followed by adrenal hypofunction (19.23%) and infrequent hypopituitarism (2.67%) (Supplementary Table S3). In addition, A meta-analysis by Barroso-Sousa et al. (80). showed that combination therapy with immune checkpoint inhibitors was more likely to involve other endocrine glands.

Type 1 diabetes caused by PD-1 inhibitors is relatively rare, with an estimated incidence of 0.2–1.4% (7–9). DKA is the most common onset (10–24), but the patient in this study presented with hyperosmolar hyperglycemic coma at the onset. Although the overall frequency of type 1 diabetes in irAEs is relatively low, PD-1 inhibitor-associated type 1 diabetes progresses rapidly to a critical illness and may endanger patients' lives if not promptly diagnosed and treated (5, 81). Clinicians should inform patients of the potential risk of diabetes with PD-1 inhibitors, train them in recognizing the symptoms of hyperglycemia and DKA, and enhance the knowledge of patients with diabetes. Currently, the Food Drug Administration–approved PD-1 inhibitors include nivolumab and pembrolizumab. Studies have shown that nivolumab is associated with an increased risk of type 1 diabetes (16, 82, 83), and the cases may increase with the more widespread use of nivolumab. Our results showed that nivolumab monotherapy had a slower progression to diabetes than pembrolizumab. In addition, patients treated with PD-1 inhibitors combined with CTLA-4 inhibitors progressed to diabetes at an earlier time.

**Figure 1:**
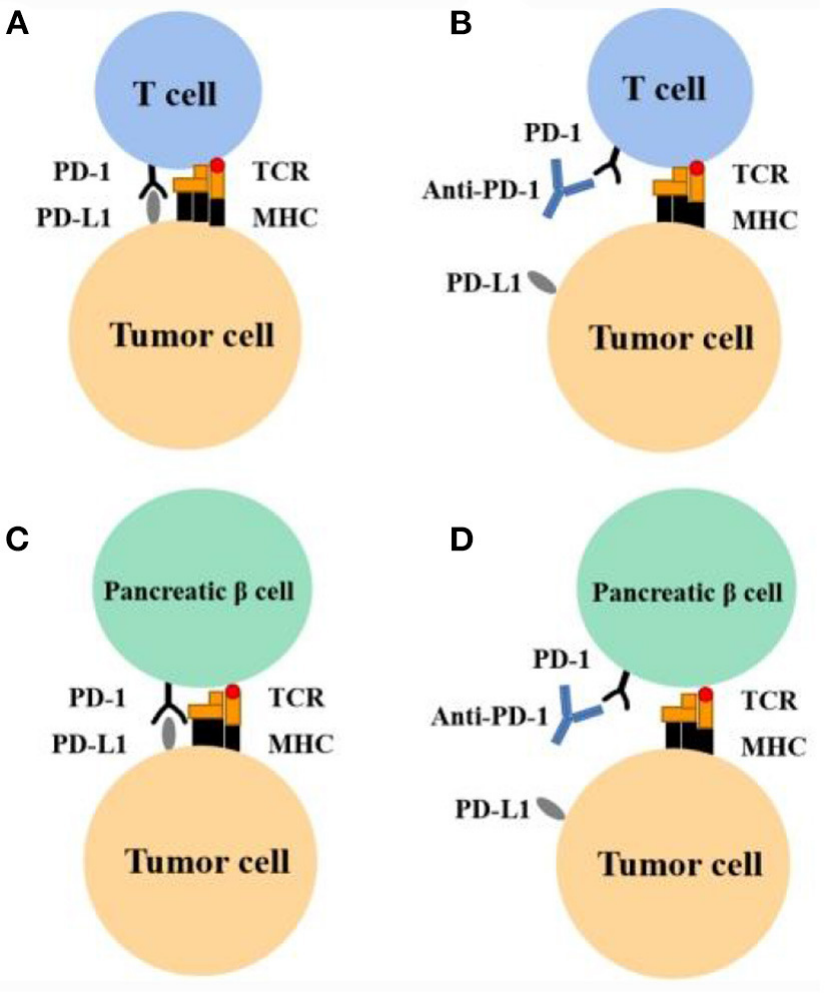
Mechanism of action of PD-1 inhibitor and hypothesis of association between PD-1 inhibitors and type 1 diabetes. **(A)** Tumor cells can inactivate T cells and evade the immune system by expressing PD-L1. This leads to the enhanced survival of tumor cells. **(B)** Anti–PD-1 can block the PD-1 receptor and restore immune response. This leads to the apoptosis of tumor cells. **(C)** Pancreatic β-cells express PD-L1 and thereby evade the immune response. **(D)** During anti–PD-1 therapy, in certain susceptible persons, T cells are activated and develop an immune response to pancreatic β-cells. MHC, major histocompatibility complex; TCR, T-cell receptor.

Based on this report and a systematic review of previous related cases, the pathogenesis of PD-1 inhibitor-associated type 1 diabetes can be summarized as follows:

**(1) Activation of proliferating T cells destroys islet β cells:** Several animal studies have demonstrated the role of PD-1 in type 1 diabetes. For example, PD-1 transgenic mice had a reduced incidence of type 1 diabetes (84), and PD-1 blockade led to the faster progression of diabetes in mice with prediabetic nonobese diabetes (NOD) (85, 86), mainly occurring through the PD-1/PD-L1 pathway; the PD-1/PD-L2 pathway is rare (87). The PD-1 expression rate on T cells (mainly CD8^+^ T cells) of patients with type 1 diabetes was lower than that of healthy persons or patients with type 2 diabetes (88, 89). PD-1 inhibitors block the PD-1/PD-L1 pathway, increasing the number of T cells or maintaining higher activity and leading to the accelerated destruction of islet β cells (87). Figure 1 provides an overview of the mechanism of action of PD-1 inhibitors and the hypothesis of an association between PD-1 inhibitors and type 1 diabetes mellitus. The PD-1/PD-L1 pathway is crucial in maintaining islet β-cell antigen tolerance, and β-cell destruction leads to faster progression to diabetes in genetically predisposed individuals (90); that is, patients with a family history of diabetes may progress to diabetes faster when treated with PD-1 inhibitors. Among the retrieved cases, two had a family history of diabetes (47, 55); they were diagnosed with diabetes after 1 cycle of treatment with a PD-1 inhibitor, with severely impaired islet function, and became dependent on insulin treatment.

**(2) Increase in insulin autoantibodies:** Currently, insulin autoantibody inhibitors correlate with PD-1 in the development of type 1 diabetes, yet the mechanism is not clear. An autoimmune diabetes NOD mouse model was established by Ansari et al. (87). found that some had autoantibody-negative diabetes while some had autoantibody-positive diabetes. The positive rate of GADA was the highest in patients with positive insulin autoantibodies. However, GADA was also found in other autoimmune endocrine diseases, such as Graves' disease (91), and hence it was not specific. In addition, Gauci et al. (92). found that the time interval between the occurrence of type 1 diabetes induced by PD-1 inhibitors was related to GADA positivity. Usui et al. (93). indicated that GADA positivity could accelerate the progression of type 1 diabetes associated with PD-1 inhibitors, which was also supported by our systematic review. In other words, patients positive for insulin autoantibodies were diagnosed with diabetes after receiving PD-1 inhibitors treatment, and the average medication period was significantly shorter than that of patients negative for autoantibodies. We considered that besides cellular immunity, humoral immunity was involved in antibody-positive patients, leading to more rapid islet failure.

**(3) HLA genotype increases susceptibility to type 1 diabetes:** HLA-specific alleles are associated with increased susceptibility to T1DM, account for 30–50% of the genetic risk of T1DM (94), especially HLA-DRB1, HLA-DQB1 and HLA-DQA1 (95). Different combinations of HLA-DRB1, DQB1, and DQA1 determine the extent of haplotypic risk. There is research shows that the most susceptible HLA haplotypes are DRB1^*^04:05–DQA1^*^03:01–DQB1^*^03:02, followed by DRB1^*^04:01–DQA1^*^03:01–DQB^*^03:02, DRB1^*^03:01–DQA1^*^05:01–DQB1^*^02:01, and DRB1^*^04:02–DQA1^*^03:01–DQB1^*^03:02 (94). Stamatouli et al. (96) reported that HLA-DR4 was dominant in patients with type 1 diabetes associated with immune checkpoint inhibitors. We also found that HLA-DR4 had the highest association rate. Several case reports have shown an established high-risk allele for T1DM (HLA-II DR4 haplotype) present in the majority of patients for whom HLA typing was available (11, 57). Additionally, a recent report indicated that the frequencies of the DRB1^*^04:05-DQB1^*^04:01 and DRB1^*^09:01-DQB1^*^03:03 haplotypes were significantly higher than the other haplotypes (97), which was in agreement with our findings. The patient in this case did not undergo HLA genetic testing, and if HLA was performed, it might be a predictor of T1DM episodes caused by camrelizumab. Further research is needed to determine whether HLA genotyping should be performed in all patients treated with PD-1 inhibitors to predict the risk of type 1 diabetes.

**Figure 2:**
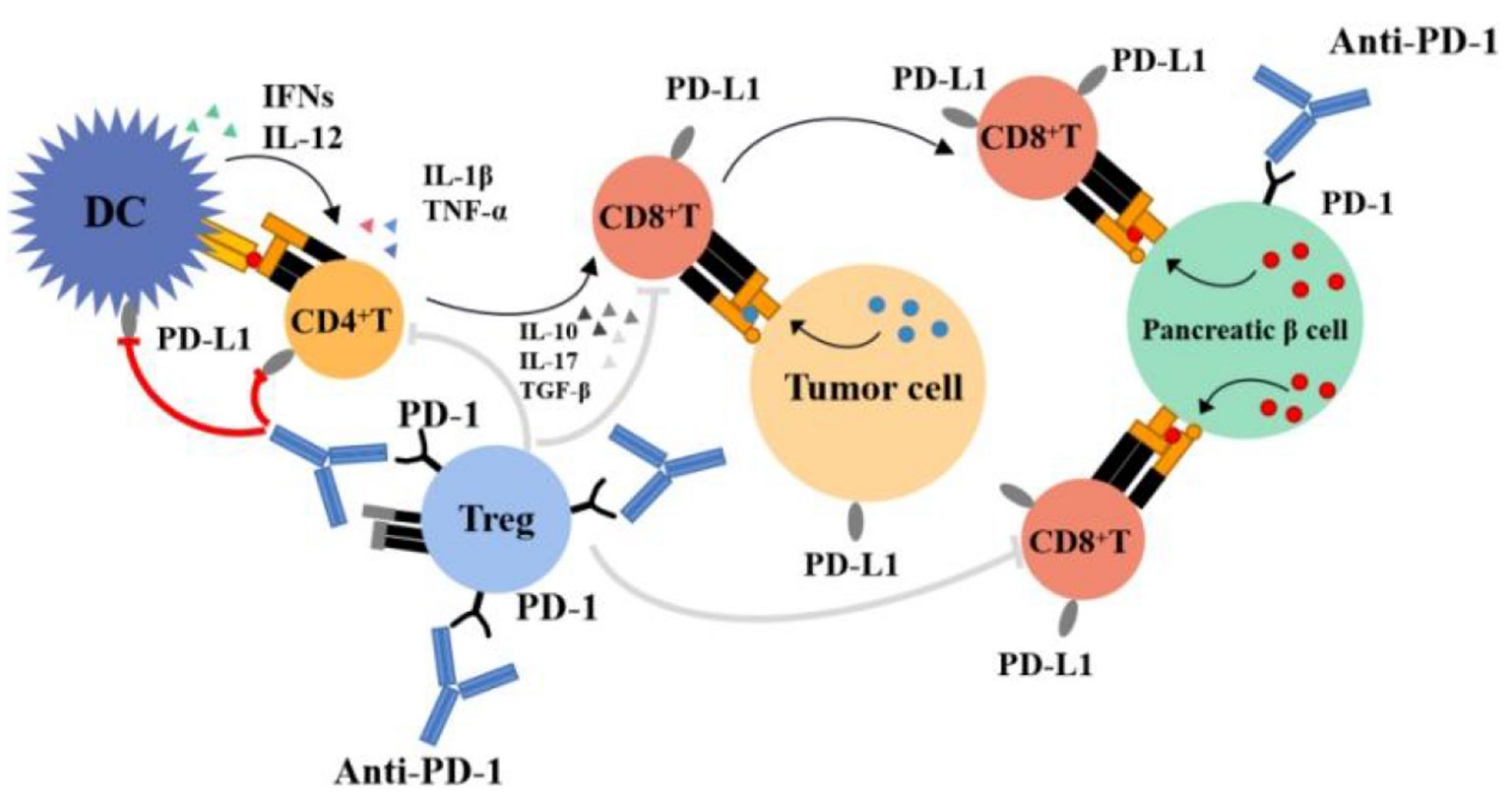
Pathophysiology of PD-1 Inhibitor–associated Type 1 Diabetes. Treg, regulatory T cells.

**(4) Increased levels of inflammatory cytokines:** Existing evidence indicates that the expression of PD-L1 can be induced by multiple inflammatory factors (98, 99). These factors mainly include interferon (IFNs), interleukin-1 β (IL-1β), interleukin-6 (IL-6), interleukin-10 (IL-10), interleukin-12 (IL-12), interleukin-17 (IL-17), transforming growth factor-β (TGF-β), and tumor necrosis factor-α (TNF-α). Colli et al. (90). suggested that IFN-γ and IFN-α upregulated the expression of PD-L1 in islet β cells of patients with diabetes to reduce susceptibility to autoimmune cells. Hence, it was speculated that islet β cells could inhibit destruction by autoimmune T cells in this way. Figure 2 illustrates the pathophysiology of PD-1 inhibitor-associated type 1 diabetes.

Different clinical phenotypes have been identified in type 1 diabetes. PD-1 inhibitor-associated diabetes is considered a novel form of type 1 diabetes that is specifically triggered by the use of PD-1 inhibitors. Reviewing the recently published literature on the subject, most agree that diabetes caused by PD-1 inhibitors is immune-mediated type 1 diabetes. There is increasing evidence that PD-1 inhibitor-associated type 1 diabetes has some specificity compared to conventional type 1 diabetes, but there are also some common diagnostic features. In addition, we refer to Wu et al. (100) summary of comparison of disease phenotypes in checkpoint inhibitor associated autoimmune diabetes versus traditional type 1 diabetes (Supplementary Table S4).

In the present case, a 70-year-old female patient denied a history of diabetes and had no obvious symptoms of hyperglycemia. At the onset of the disease, the plasma glucose level significantly increased, islet failure occurred, and the patient was dependent on insulin treatment. Combined with previous reports of related cases (10–24), the characteristics of PD-1 inhibitor-associated type 1 diabetes can be summarized as follows: (1) Late-onset age. More common in elderly people; (2) Fast islet failure. Most patients have C-peptide <0.1 ng/mL at onset; (3) Diverse clinical manifestations. Including polydipsia, polydipsia, polyuria, nausea, vomiting, dizziness, fatigue, abdominal pain, diarrhea, and even coma; (4) Potential combination with other endocrine gland dysfunctions. Which is often associated with thyroid dysfunction.

PD-1 inhibitor-associated type 1 diabetes progresses rapidly and causes critical illness. If the diagnosis and treatment are not timely, it may endanger the patient's life. Clinicians should inform patients about the potential risks of PD-1 inhibitors in diabetes and the ways to identify hyperglycemia and DKA symptoms, enhance diabetes-related knowledge, and regularly detect plasma glucose level, urine glucose level, blood ketones, urine ketone, electrolyte, arterial blood gas, and so forth. Moreover, most reports stated that there were no remissions of type 1 diabetes regardless of cessation of PD-1 inhibitor treatment (64). That is, stopping the PD-1 inhibitor will not influence the recovery of β-cells, requiring long-term insulin therapy (101). Whether to continue PD-1 inhibitor treatment once glycemic control has been attained has not yet been established. The American Society of Clinical Oncology and National Comprehensive Cancer Network guidelines recommend withholding therapy until glucose control is achieved (102). This patient continued to receive camrelizumab after stable glycemic control.

In addition, PD-1 inhibitors are also used for treating patients with type 2 diabetes complicated with tumors. We reviewed the cases of six patients with a history of type 2 diabetes who developed DKA after two treatment cycles with PD-1 inhibitors (11, 27, 36, 44, 68, 72) and had positive insulin autoantibodies and low C-peptide levels. Whether such patients are more sensitive to immune checkpoint inhibitors needs further exploration.

## Conclusion

In summary, as PD-1 inhibitors are widely used by patients with cancer, the reports of type 1 diabetes should attract the attention of clinicians. Further, they should help improve the recognition of hyperglycemia or DKA symptoms in patients, necessitating the close follow-up of patients during treatment, regular monitoring of plasma glucose level, prompt detection, and correct diagnosis and treatment of diabetes. Further, novel biomarkers of susceptibility should be identified to better guide drug treatment in the future.

## Data availability statement

The original contributions presented in the study are included in the article/Supplementary material, further inquiries can be directed to the corresponding author.

## Ethics statement

Written informed consent was obtained from the individual(s) for the publication of any potentially identifiable images or data included in this article.

## Author contributions

All authors listed have made a substantial, direct, and intellectual contribution to the work and approved it for publication.

## Conflict of interest

The authors declare that the research was conducted in the absence of any commercial or financial relationships that could be construed as a potential conflict of interest.

## Publisher's note

All claims expressed in this article are solely those of the authors and do not necessarily represent those of their affiliated organizations, or those of the publisher, the editors and the reviewers. Any product that may be evaluated in this article, or claim that may be made by its manufacturer, is not guaranteed or endorsed by the publisher.
