# Supplementary material for: PD-1 inhibitor-associated type 1 diabetes: A case report and systematic review
Source: Front Public Health. 2022 Aug 5;10:885001. doi: 10.3389/fpubh.2022.885001 (PMC9389003; doi:10.3389/fpubh.2022.885001)
Supplement: Supplementary file 1 [file Table_1.DOCX]

**Supplementary Table 1|** Islet autoantibodies, and HLA genotypes

|  | **Authors** | **Age/y** | **sex** | **Pancreas autoantibodies** | **HLA typing** |
| --- | --- | --- | --- | --- | --- |
| 2022 | J. Yang et al(1) | 83 | F | negative | HLA-DRB1*04:01-14:03, DPB1*02:01-02:01, DQB1*03:01-03:02 |
| 2022 | S. Hatayama et al(2) | 74 | M | negative | HLA-DRB1 *13:02, DQB1 *06:04 |
| 2022 | H. Alchalabi et al(3) | 56 | M | - | - |
| 2022 | T. Sato et al(4) | 43 | M | negative | HLA-DRB1*11:01, DQB1*03:01:01, DRB1*13:02:01, DQB1*06:04:01 |
| 2022 | N. Ganta et al(5) | 62 | F | - | - |
| 2021 | A. A. Glibka et al(6) | 34 | M | GADA、ICA | - |
| 2021 | S. Baroud et al(7) | 67 | F | GADA | - |
| 2021 | A. O. Saleh et al(8) | 63 | M | negative | HLA-DRB1 |
| 2021 | K. Sankar et al(9) | 85 | F | negative | - |
| 2021 | Huang, X et al(10) | 59 | M | negative | - |
| 2021 | K. Yaura et al(11) | 60 | F | negative | HLA-DRB1*11:01:01, DRB1*12:01:01, DQB1*03:01:01 |
| 2021 | F. Kikuchi et al(12) | 62 | M | negative | - |
| 2021 | L. Boswell et al(13) | 51 | M | negative | HLA- DRB1*01:01，DRB1*12:01 |
| 2021 | L. Wu et al(14) | 38 | F | negative | - |
| 2021 | A. Hernandez et al(15) | 67 | M | - | - |
| 2021 | S. K. Kedzior et al(16) | 51 | M | GADA | - |
| 2021 | S. Jessel et al(17) | 60 | M | - | - |
| 2021 | H. Yamaguchi et al(18) | 55 | M | GADA、IA-2A | HLA-DRB1*08:03-09:01, DQA1*01:03-03:01, DQB1*03:03-06:01 |
| 2021 | K. Oldfield et al(19) | 76 | M | - | - |
| 2020 | Wen Liang et al (20) | 56 | M | negative | HLA-A*02:01-24:03, DRB1*12:01-12:02, DQB1*05:03-03:01, DQA1*01:04-06:01 |
| 2020 | Kyriacou et al (21) | 68 | F | negative | - |
| 2020 | J. A. Cuenca et al (22) | 62 | M | negative | - |
| 2020 | D. Keerty et al (23) | 49 | F | negative | - |
| 2020 | A. Kichloo et al (24) | 77 | F | negative | - |
| 2020 | R. A. Samoa et al(25) | 12 | M | IAA, IA-2A | HLA-DR4 |
| 2020 | K. Kusuki et al(26) | 72 | M | negative | HLA -DRB1*09:01-DQB1*03:03 |
| 2020 | S. Marshall et al(27) | 70 | M | negative | - |
| 2020 | S. Kurihara et al(28) | 48 | M | negative | HLA-DRB1*04:05 |
| 2020 | M. Miyauchi et al(29) | 79 | M | negative | HLA -DRB1*09:01-DQB1*03:03 |
| 2020 | V. Singh et al(30) | 65 | F | negative | - |
| 2020 | N. Ohara et al(31) | 70 | M | IA-2A | HLA-DRB1*04:03-08:03， DQB1*03:02-06:01，DQA1*01:03-03:01, DPB1*02:01-02:02 |
| 2020 | W. Haque et al(32) | 78 | M | GADA | - |
| 2020 | T. Porntharukchareon et al(33) | 70 | M | negative | - |
| 2020 | M. S. Hughes(34) | 48 | M | GADA | HLA-DRB1*03, DQA1*05:01, DQB1*02 |
| 2019 | O. A. Hakami et al(35) | 52 | M | negative | - |
| 2019 | M. Zezza et al(36) | 60 | M | GADA、ICA、 IA-2A | - |
| 2019 | M. Zezza et al(36) | 80 | F | GADA、ICA | - |
| 2019 | N. Yamamoto et al(37) | 77 | M | negative | HLA-DRB1* 09:01:02-12:01:01, DQB1* 03:01:01-03:03:02,  DPB1* 05:01:01, DQA1*03:02-05:05 |
| 2019 | H. M. A. Abdullah et al(38) | 68 | M | negative | - |
| 2019 | C. Sakaguchi et al(39) | 68 | F | negative | HLA-DRB1* 09:01-DQB1* 03:03 |
| 2019 | R. Edahiro et al(40) | 61 | M | negative | - |
| 2019 | Y. Tohi et al(41) | 75 | M | negative | - |
| 2019 | T. Alrifai et al(42) | 69 | M | GADA | - |
| 2019 | J. M. K. de Filette et al(43) | 61 | M | GADA | HLA-DRB1*04, DQA1*03:01, DQB1*03:02 |
| 2019 | D. Saito et al(44) | 82 | M | negative | HLA-DRB1*12:01 |
| 2018 | E. S. Scott et al(45) | 58 | M | negative | - |
| 2018 | S. Li et al(46) | 67 | M | negative | - |
| 2018 | Akturk HK et al(47) | 62 | M | negative | HLA-DRB * 03:01，DQA * 05:01，DQB * 02:01（DR3-DQ2） |
| 2018 | N. Chokr et al(48) | 61 | M | negative | negative |
| 2018 | F. Gunawan et al(49) | 52 | M | negative | - |
| 2018 | S. Lee et al(50) | 67 | M | GADA | - |
| 2018 | K. Sakurai et al(51) | 68 | F | negative | HLA-DRB1* 09:01-DQB1* 03:03 |
| 2018 | M. Shiba et al(52) | 80 | F | negative | HLA-DR4，DR12 |
| 2018 | P. Tzoulis et al(53) | 56 | F | GADA | negative |
| 2018 | K. Matsumura et al(54) | 68 | M | negative | HLA- DRB1*09:01-DRB1*15:02 |
| 2018 | A. A. Zaied et al(55) | 70 | M | negative | - |
| 2017 | R. Capitao et al(56) | 74 | F | GADA | HLA-DRB1*04 |
| 2017 | P. N. Changizzadeh et al(57) | 42 | M | negative | - |
| 2017 | J. L. Godwin et al(58) | 34 | M | GADA、IA-2A、ZnT8A | HLA-D09：CTZ，09：CTZ (DR9) |
| 2017 | Kapke J(59) | 83 | M | GADA | HLA-DRB1*08:11, DQB1*03:04，DQA1* 04:05 |
| 2017 | R. Kumagai et al(60) | 73 | M | negative | HLA-DB1*09:01-DQB1*03:03，DRB1*01:01-DQB1*05:01 |
| 2017 | L. Li et al(61) | 63 | M | GADA | - |
| 2017 | L. Marchand et al(62) | 55 | M | negative | - |
| 2016 | M. Okamoto et al(63) | 55 | F | negative | HLA-DRB1*04:05，DQB1*04:01 |
| 2016 | J. Aleksova et al(64) | 60 | M | negative | - |
| 2016 | M. A. Humayun et al(65) | 55 | M | negative | - |
| 2016 | Y. Miyoshi et al(66) | 66 | F | negative | HLA-DRB1*11:01-13:02:01，DQB1*03:01:01-06:04:01 |
| 2016 | J. R. Lowe et al (67) | 54 | M | GADA | HLA-DQB1*06:02 |
| 2015 | C. Gaudy et al(68) | 44 | F | negative | - |
| 2015 | J. Hughes et al(69) | 55 | F | negative | HLA-A2.1, DR4 |
| 2015 | J. Hughes et al(69) | 83 | F | GADA | HLA-A2.1, DR4 |
| 2015 | J. Hughes et al(69) | 63 | M | GADA、ICA、 IAA | HLA-A2.1, DR4 |
| 2015 | J. Hughes et al(69) | 58 | M | GADA | HLA-A2.1, DR4 |
| 2015 | J. Hughes et al(69) | 64 | F | negative | HLA-DR4 |
| 2020 | Cuiping Lin | 70 | F | IA-2A | - |

“-”: Undetected.

**Reference**

1. Yang J, Wang Y, Tong XM. Sintilimab-induced autoimmune diabetes: A case report and review of the literature. World J Clin Cases. 2022;10(4):1263-77.

2. Hatayama S, Kodama S, Kawana Y, Otake S, Sato D, Horiuchi T, et al. Two cases with fulminant type 1 diabetes that developed long after cessation of immune checkpoint inhibitor treatment. J Diabetes Investig. 2022.

3. Alchalabi H, Albustani S, Fareen N, Udongwo N, Chaughtai S, Holland S. An Unusual Etiology of Hypothyroidism and New-Onset Insulin-Dependent Diabetes: A Rare Side Effect of Nivolumab. Cureus. 2022;14(4):e24463.

4. Sato T, Kodama S, Kaneko K, Imai J, Katagiri H. Type 1 Diabetes Mellitus Associated with Nivolumab after Second SARS-CoV-2 Vaccination, Japan. Emerg Infect Dis. 2022;28(7).

5. Ganta N, Alnabwani D, Keating S, Patel V, Bommu VJL, Dawoud R, et al. Rare Adverse Events Related to Nivolumab, an Immune Checkpoint Inhibitor: A Case Series. Cureus. 2022;14(2):e22070.

6. Glibka AA, Mel Nichenko GA, Mikhina MS, Mazurina NV, Kharkevich GY. [Development of destructive thyroiditis and diabetes mellitus after three injections of pembrolizumab for skin melanoma]. Probl Endokrinol (Mosk). 2021;67(2):20-7.

7. Baroud S, Mirza L. New-Onset Type 1 Diabetes Mellitus After Treatment With Nivolumab for Melanoma. Cureus. 2021;13(10):e18679.

8. Saleh AO, Taha R, Mohamed SFA, Bashir M. Hyperosmolar Hyperglycaemic State and Diabetic Ketoacidosis in Nivolumab-Induced Insulin-Dependent Diabetes Mellitus. Eur J Case Rep Intern Med. 2021;8(8):002756.

9. Sankar K, Macfarlane M, Cooper O, Falk J. Pembrolizumab-Induced Diabetic Ketoacidosis: A Review of Critical Care Case. Cureus. 2021;13(10):e18983.

10. Huang X, Yang M, Wang L, Li L, Zhong X. Sintilimab induced diabetic ketoacidosis in a patient with small cell lung cancer: A case report and literature review. Medicine (Baltimore). 2021;100(19):e25795.

11. Yaura K, Sakurai K, Niitsuma S, Sato R, Takahashi K, Arihara Z. Fulminant Type 1 Diabetes Mellitus Developed about Half a Year after Discontinuation of Immune Checkpoint Inhibitor Combination Therapy with Nivolumab and Ipilimumab: A Case Report. Tohoku J Exp Med. 2021;254(4):253-6.

12. Kikuchi F, Saheki T, Imachi H, Kobayashi T, Fukunaga K, Ibata T, et al. Nivolumab-induced hypophysitis followed by acute-onset type 1 diabetes with renal cell carcinoma: a case report. J Med Case Rep. 2021;15(1):214.

13. Boswell L, Casals G, Blanco J, Jiménez A, Aya F, de Hollanda A, et al. Onset of fulminant type 1 diabetes mellitus following hypophysitis after discontinuation of combined immunotherapy. A case report. J Diabetes Investig. 2021;12(12):2263-6.

14. Wu L, Li B. A Case of Severe Diabetic Ketoacidosis Associated with Pembrolizumab Therapy in a Patient with Metastatic Melanoma. Diabetes Metab Syndr Obes. 2021;14:753-7.

15. Hernandez A, Zeidan B, Jr., Desai P, Frunzi J. Diabetic Ketoacidosis Secondary to New Onset Type 1 Diabetes Mellitus Related to Pembrolizumab Therapy. Cureus. 2021;13(2):e13302.

16. Kedzior SK, Jacknin G, Hudler A, Mueller SW, Kiser TH. A Severe Case of Diabetic Ketoacidosis and New-Onset Type 1 Diabetes Mellitus Associated with Anti-Glutamic Acid Decarboxylase Antibodies Following Immunotherapy with Pembrolizumab. Am J Case Rep. 2021;22:e931702.

17. Jessel S, Austin M, Kluger HM. Mycophenolate as Primary Treatment for Immune Checkpoint Inhibitor Induced Acute Kidney Injury in a Patient with Concurrent Immunotherapy-Associated Diabetes: A Case Report. Clin Oncol Case Rep. 2021;4(1).

18. Yamaguchi H, Miyoshi Y, Uehara Y, Fujii K, Nagata S, Obata Y, et al. Case of slowly progressive type 1 diabetes mellitus with drastically reduced insulin secretory capacity after immune checkpoint inhibitor treatment for advanced renal cell carcinoma. Diabetol Int. 2021;12(2):234-40.

19. Oldfield K, Jayasinghe R, Niranjan S, Chadha S. Immune checkpoint inhibitor-induced takotsubo syndrome and diabetic ketoacidosis: rare reactions. BMJ Case Rep. 2021;14(2).

20. Wen L, Zou X, Chen Y, Bai X, Liang T. Sintilimab-Induced Autoimmune Diabetes in a Patient With the Anti-tumor Effect of Partial Regression. Front Immunol. 2020;11:2076.

21. Kyriacou A, Melson E, Chen W, Kempegowda P. Is immune checkpoint inhibitor-associated diabetes the same as fulminant type 1 diabetes mellitus? Clin Med (Lond). 2020;20(4):417-23.

22. Cuenca JA, Laserna A, Reyes MP, Nates JL, Botz GH. Critical Care Admission of an HIV Patient with Diabetic Ketoacidosis Secondary to Pembrolizumab. Case Rep Crit Care. 2020;2020:8671530.

23. Keerty D, Das M, Hallanger-Johnson J, Haynes E. Diabetic Ketoacidosis: An Adverse Reaction to Immunotherapy. Cureus. 2020;12(9):e10632.

24. Kichloo A, Albosta MS, McMahon S, Movsesian K, Wani F, Jamal SM, et al. Pembrolizumab-Induced Diabetes Mellitus Presenting as Diabetic Ketoacidosis in a Patient With Metastatic Colonic Adenocarcinoma. J Investig Med High Impact Case Rep. 2020;8:2324709620951339.

25. Samoa RA, Lee HS, Kil SH, Roep BO. Anti-PD-1 Therapy-Associated Type 1 Diabetes in a Pediatric Patient With Relapsed Classical Hodgkin Lymphoma. Diabetes Care. 2020;43(9):2293-5.

26. Kusuki K, Suzuki S, Mizuno Y. Pembrolizumab-induced fulminant type 1 diabetes with C-peptide persistence at first referral. Endocrinol Diabetes Metab Case Rep. 2020;2020.

27. Marshall S, Kizuki A, Kitaoji T, Imada H, Kato H, Hosoda M, et al. Type 1 Diabetes, ACTH Deficiency, and Hypothyroidism Simultaneously Induced by Nivolumab Therapy in a Patient with Gastric Cancer: A Case Report. Case Rep Oncol. 2020;13(3):1185-90.

28. Kurihara S, Oikawa Y, Nakajima R, Satomura A, Tanaka R, Kagamu H, et al. Simultaneous development of Graves' disease and type 1 diabetes during anti-programmed cell death-1 therapy: A case report. J Diabetes Investig. 2020;11(4):1006-9.

29. Miyauchi M, Toyoda M, Zhang J, Hamada N, Yamawaki T, Tanaka J, et al. Nivolumab-induced fulminant type 1 diabetes with precipitous fall in C-peptide level. J Diabetes Investig. 2020;11(3):748-9.

30. Singh V, Chu Y, Gupta V, Zhao CW. A Tale of Immune-Related Adverse Events With Sequential Trials of Checkpoint Inhibitors in a Patient With Metastatic Renal Cell Carcinoma. Cureus. 2020;12(6):e8395.

31. Ohara N, Kobayashi M, Ikeda Y, Hoshi T, Morita S, Kanefuji T, et al. Non-insulin-dependent Diabetes Mellitus Induced by Immune Checkpoint Inhibitor Therapy in an Insulinoma-associated Antigen-2 Autoantibody-positive Patient with Advanced Gastric Cancer. Intern Med. 2020;59(4):551-6.

32. Haque W, Ahmed SR, Zilbermint M. Nivolumab-induced autoimmune diabetes mellitus and hypothyroidism in a patient with rectal neuroendocrine tumor. J Community Hosp Intern Med Perspect. 2020;10(4):338-9.

33. Porntharukchareon T, Tontivuthikul B, Sintawichai N, Srichomkwun P. Pembrolizumab- and ipilimumab-induced diabetic ketoacidosis and isolated adrenocorticotropic hormone deficiency: a case report. J Med Case Rep. 2020;14(1):171.

34. Hughes MS, Pietropaolo M, Vasudevan MM, Marcelli M, Nguyen H. Checking the Checkpoint Inhibitors: A Case of Autoimmune Diabetes After PD-1 Inhibition in a Patient with HIV. J Endocr Soc. 2020;4(12):bvaa150.

35. Hakami OA, Ioana J, Ahmad S, Tun TK, Sreenan S, McDermott JH. A case of pembrolizumab-induced severe DKA and hypothyroidism in a patient with metastatic melanoma. Endocrinol Diabetes Metab Case Rep. 2019;2019.

36. Zezza M, Kosinski C, Mekoguem C, Marino L, Chtioui H, Pitteloud N, et al. Combined immune checkpoint inhibitor therapy with nivolumab and ipilimumab causing acute-onset type 1 diabetes mellitus following a single administration: two case reports. BMC Endocr Disord. 2019;19(1):144.

37. Yamamoto N, Tsurutani Y, Katsuragawa S, Kubo H, Sunouchi T, Hirose R, et al. A Patient with Nivolumab-related Fulminant Type 1 Diabetes Mellitus whose Serum C-peptide Level Was Preserved at the Initial Detection of Hyperglycemia. Intern Med. 2019;58(19):2825-30.

38. Abdullah HMA, Elnair R, Khan UI, Omar M, Morey-Vargas OL. Rapid onset type-1 diabetes and diabetic ketoacidosis secondary to nivolumab immunotherapy: a review of existing literature. BMJ Case Rep. 2019;12(8).

39. Sakaguchi C, Ashida K, Yano S, Ohe K, Wada N, Hasuzawa N, et al. A case of nivolumab-induced acute-onset type 1 diabetes mellitus in melanoma. Curr Oncol. 2019;26(1):e115-e8.

40. Edahiro R, Ishijima M, Kurebe H, Nishida K, Uenami T, Kanazu M, et al. Continued administration of pembrolizumab for adenocarcinoma of the lung after the onset of fulminant type 1 diabetes mellitus as an immune-related adverse effect: A case report. Thorac Cancer. 2019;10(5):1276-9.

41. Tohi Y, Fujimoto K, Suzuki R, Suzuki I, Kubota M, Kawakita M. Fulminant type 1 diabetes mellitus induced by pembrolizumab in a patient with urothelial carcinoma: A case report. Urol Case Rep. 2019;24:100849.

42. Alrifai T, Ali FS, Saleem S, Ruiz DCM, Rifai D, Younas S, et al. Immune Checkpoint Inhibitor Induced Diabetes Mellitus Treated with Insulin and Metformin: Evolution of Diabetes Management in the Era of Immunotherapy. Case Rep Oncol Med. 2019;2019:8781347.

43. de Filette JMK, Pen JJ, Decoster L, Vissers T, Bravenboer B, Van der Auwera BJ, et al. Immune checkpoint inhibitors and type 1 diabetes mellitus: a case report and systematic review. Eur J Endocrinol. 2019;181(3):363-74.

44. Saito D, Oikawa Y, Yano Y, Ikegami Y, Satomura A, Isshiki M, et al. Detailed Time Course of Decline in Serum C-Peptide Levels in Anti-Programmed Cell Death-1 Therapy-Induced Fulminant Type 1 Diabetes. Diabetes Care. 2019;42(3):e40-e1.

45. Scott ES, Long GV, Guminski A, Clifton-Bligh RJ, Menzies AM, Tsang VH. The spectrum, incidence, kinetics and management of endocrinopathies with immune checkpoint inhibitors for metastatic melanoma. Eur J Endocrinol. 2018;178(2):173-80.

46. Li S, Zhang Y, Sun Z, Hu J, Fang C. Anti-PD-1 pembrolizumab induced autoimmune diabetes in Chinese patient: A case report. Medicine (Baltimore). 2018;97(45):e12907.

47. Akturk HK, Alkanani A, Zhao Z, Yu L, Michels AW. PD-1 Inhibitor Immune-Related Adverse Events in Patients With Preexisting Endocrine Autoimmunity. J Clin Endocrinol Metab. 2018;103(10):3589-92.

48. Chokr N, Farooq H, Guadalupe E. Fulminant Diabetes in a Patient with Advanced Melanoma on Nivolumab. Case Rep Oncol Med. 2018;2018:8981375.

49. Gunawan F, George E, Roberts A. Combination immune checkpoint inhibitor therapy nivolumab and ipilimumab associated with multiple endocrinopathies. Endocrinol Diabetes Metab Case Rep. 2018;2018.

50. Lee S, Morgan A, Shah S, Ebeling PR. Rapid-onset diabetic ketoacidosis secondary to nivolumab therapy. Endocrinol Diabetes Metab Case Rep. 2018;2018.

51. Sakurai K, Niitsuma S, Sato R, Takahashi K, Arihara Z. Painless Thyroiditis and Fulminant Type 1 Diabetes Mellitus in a Patient Treated with an Immune Checkpoint Inhibitor, Nivolumab. Tohoku J Exp Med. 2018;244(1):33-40.

52. Shiba M, Inaba H, Ariyasu H, Kawai S, Inagaki Y, Matsuno S, et al. Fulminant Type 1 Diabetes Mellitus Accompanied by Positive Conversion of Anti-insulin Antibody after the Administration of Anti-CTLA-4 Antibody Following the Discontinuation of Anti-PD-1 Antibody. Intern Med. 2018;57(14):2029-34.

53. Tzoulis P, Corbett RW, Ponnampalam S, Baker E, Heaton D, Doulgeraki T, et al. Nivolumab-induced fulminant diabetic ketoacidosis followed by thyroiditis. Endocrinol Diabetes Metab Case Rep. 2018;2018.

54. Matsumura K, Nagasawa K, Oshima Y, Kikuno S, Hayashi K, Nishimura A, et al. Aggravation of diabetes, and incompletely deficient insulin secretion in a case with type 1 diabetes-resistant human leukocyte antigen DRB1*15:02 treated with nivolumab. J Diabetes Investig. 2018;9(2):438-41.

55. Zaied AA, Akturk HK, Joseph RW, Lee AS. New-onset insulin-dependent diabetes due to nivolumab. Endocrinol Diabetes Metab Case Rep. 2018;2018.

56. Capitao R, Bello C, Fonseca R, Saraiva C. New onset diabetes after nivolumab treatment. BMJ Case Rep. 2018;2018.

57. Changizzadeh PN, Mukkamalla SKR, Armenio VA. Combined checkpoint inhibitor therapy causing diabetic ketoacidosis in metastatic melanoma. J Immunother Cancer. 2017;5(1):97.

58. Godwin JL, Jaggi S, Sirisena I, Sharda P, Rao AD, Mehra R, et al. Nivolumab-induced autoimmune diabetes mellitus presenting as diabetic ketoacidosis in a patient with metastatic lung cancer. J Immunother Cancer. 2017;5:40.

59. Kapke J, Shaheen Z, Kilari D, Knudson P, Wong S. Immune Checkpoint Inhibitor-Associated Type 1 Diabetes Mellitus: Case Series, Review of the Literature, and Optimal Management. Case Rep Oncol. 2017;10(3):897-909.

60. Kumagai R, Muramatsu A, Nakajima R, Fujii M, Kaino K, Katakura Y, et al. Acute-onset type 1 diabetes mellitus caused by nivolumab in a patient with advanced pulmonary adenocarcinoma. J Diabetes Investig. 2017;8(6):798-9.

61. Li L, Masood A, Bari S, Yavuz S, Grosbach AB. Autoimmune Diabetes and Thyroiditis Complicating Treatment with Nivolumab. Case Rep Oncol. 2017;10(1):230-4.

62. Marchand L, Paulus V, Fabien N, Pérol M, Thivolet C, Vouillarmet J, et al. Nivolumab-Induced Acute Diabetes Mellitus and Hypophysitis in a Patient with Advanced Pulmonary Pleomorphic Carcinoma with a Prolonged Tumor Response. J Thorac Oncol. 2017;12(11):e182-e4.

63. Okamoto M, Okamoto M, Gotoh K, Masaki T, Ozeki Y, Ando H, et al. Fulminant type 1 diabetes mellitus with anti-programmed cell death-1 therapy. J Diabetes Investig. 2016;7(6):915-8.

64. Aleksova J, Lau PK, Soldatos G, McArthur G. Glucocorticoids did not reverse type 1 diabetes mellitus secondary to pembrolizumab in a patient with metastatic melanoma. BMJ Case Rep. 2016;2016.

65. Humayun MA, Poole R. A case of multiple immune toxicities from Ipilimumab and pembrolizumab treatment. Hormones (Athens). 2016;15(2):303-6.

66. Miyoshi Y, Ogawa O, Oyama Y. Nivolumab, an Anti-Programmed Cell Death-1 Antibody, Induces Fulminant Type 1 Diabetes. Tohoku J Exp Med. 2016;239(2):155-8.

67. Lowe JR, Perry DJ, Salama AK, Mathews CE, Moss LG, Hanks BA. Genetic risk analysis of a patient with fulminant autoimmune type 1 diabetes mellitus secondary to combination ipilimumab and nivolumab immunotherapy. J Immunother Cancer. 2016;4:89.

68. Gaudy C, Clévy C, Monestier S, Dubois N, Préau Y, Mallet S, et al. Anti-PD1 Pembrolizumab Can Induce Exceptional Fulminant Type 1 Diabetes. Diabetes Care. 2015;38(11):e182-3.

69. Hughes J, Vudattu N, Sznol M, Gettinger S, Kluger H, Lupsa B, et al. Precipitation of autoimmune diabetes with anti-PD-1 immunotherapy. Diabetes Care. 2015;38(4):e55-7.
