# Supplementary material for: PD-1 inhibitor-associated type 1 diabetes: A case report and systematic review
Source: Front Public Health. 2022 Aug 5;10:885001. doi: 10.3389/fpubh.2022.885001 (PMC9389003; doi:10.3389/fpubh.2022.885001)
Supplement: Supplementary file 2 [file Table_2.docx]

**Supplementary Table 2|** Case-related data

| **Characteristic** | **All cases（n=75）** |
| --- | --- |
| **Age, years**  Median (range) | 63 (12-85) |
| **Gender**  Female/male | 23/52 |
| **Tumor types**  Melanoma  NSCLC  RCC  Others | 36.00% (27/75)  16.00% (12/75)  12.00% (9/75)  36.00% (27/75) |
| **Immune checkpoint inhibitor**  Carrelizumab  Sintilimab  Nivolumab  Pembrolizumab  Ipilimumab and Nivolumab  Ipilimumab and Pembrolizumab | 1.33% (1/75)  4.00% (3/75)  44.00% (33/75)  26.67% (19/75)  20.00% (15/75)  5.33% (4/75) |
| **Time-to-diagnosis in cycles (range)** | 6.11 (1-28) |
| **Glycemia(mg/dL ), median (range)** | 656 (271-1298) |
| **HbA1c (%), median (range)** | 7.85 (6.1-11.1) |
| **With/without DKA** | 57/18 |
| **Low-C-peptide at diagnosis (<0.1 ng/ml)** | 50.67% (38/75) |
| **Positive pancreas autoantibodies** | 30.67% (23/75) |
| **Type of pancreas autoantibodies**  GADA  ICA  IA-2A  IAA  ZnT8A | 86.96% (20/23)  17.39% (4/23)  26.09% (6/23)  8.70% (2/23)  4.35% (1/23) |
| **Pre/family history of diabetes** | 12% (9/75) |
| **HLA analysis**  HLA-DR4 | 46.67% (35/75)  37.14% (13/35) |

NSCLC, Non-small cell lung cancer; RCC, renal cell carcinoma; DKA, diabetes ketoacidosis; HLA, human leukocyte antigen.
