# Supplementary material for: PD-1 inhibitor-associated type 1 diabetes: A case report and systematic review
Source: Front Public Health. 2022 Aug 5;10:885001. doi: 10.3389/fpubh.2022.885001 (PMC9389003; doi:10.3389/fpubh.2022.885001)
Supplement: Supplementary file 3 [file Table_3.docx]

**Supplementary Table 3|** Monotherapy and combination

| **Immune checkpoint inhibitor** | Nivolumab | Pembrolizumab | Ipilimumab and Nivolumab | Ipilimumab and Pembrolizumab |
| --- | --- | --- | --- | --- |
| **Time-to-diagnosis in cycles,** **median (range)** | 6.47 (1-28) | 6.5 (1-16) | 4.47 (1-20) | 4.75 (2-9) |
| **Glucose, mg/dL, median (range)** | 612 (271-1298) | 727 (275.4-1194) | 776 (330-1211) | 674 (486-794) |
| **HbA1c(%), median (range)** | 7.83 (7-10.2) | 8.00 (6.1-11) | 7.45 (6.5-9.8) | 7.8 (6.5-10.6) |
| **Cases** | 33 | 19 | 15 | 4 |
| **Pancreatic autoantibodies-positive** | 42.42% (14/33) | 26.32% (5/19) | 26.67 (4/15) | 0 |
| **With/without DKA** | 21 vs 12 | 17 vs 2 | 12 vs 3 | 4 vs 0 |
| **Endocrinopathy**  Thyroid (n=26) | 50% (13/26) | 19.23% (5/26) | 19.23% (5/26) | 11.54% (3/26) |
| Adrenal (n=6) | 50% (3/6) | 0 | 33.33% (2/6) | 16.67% (1/6) |
| Pituitary (n=2) | 0 | 0 | 100%(2/2) | 0 |
