# Supplementary material for: PD-1 inhibitor-associated type 1 diabetes: A case report and systematic review
Source: Front Public Health. 2022 Aug 5;10:885001. doi: 10.3389/fpubh.2022.885001 (PMC9389003; doi:10.3389/fpubh.2022.885001)
Supplement: Supplementary file 4 [file Table_4.docx]

**Supplementary Table 4｜**Comparison of the disease phenotype of PD-1 inhibitor-associated type 1 diabetes to traditional type 1 diabetes mellitus(T1DM)

|  | **PD-1 inhibitor-associated type 1 diabetes** | **Type 1 diabetes mellitus** |
| --- | --- | --- |
| **Presentation** | PD-1 inhibitor-associated type 1 diabetes is most diagnosed in older. DKA in 76% at presentation. 41.33% have a comorbid irAE, most common being thyroid (34.67%). The accompanying hyperglycemia can be more severe than in conventional T1DM and the onset is abrupt[1]. | T1DM is most commonly diagnosed in children and young adults. DKA in 19.8% childhood and adolescence at presentation [2]. |
| **Clinical course** | No spontaneous remission phase or "honeymoon phase". Overt insulin deficiency and low C-peptide at presentation[3]. | Patients with T1DM present a "honeymoon phase" with partial recovery of islet β-cell function[4]. |
| **Autoantibodies** | Only 32.86% of PD-1 inhibitor-associated type 1 diabetes individuals are positive for any islet autoantibody; anti‐GAD65 is by far the most common at 86.96%. | Over 90% of T1DM patients have developed at least one positive autoantibody by the time of diagnosis[5]. |
| **Genetic predisposition** | 61% with T1DM susceptibility haplotype, 16% with T1DM protective haplotype[6]. | T1DM susceptible haplotypes in 90%[7]. |
| **Exocrine pancreas involvement** | Pancreatic enzymes elevated, pancreatic atrophy on imaging[1]. | Lower lipase vs normal controls except in fulminant phenotype, reduced pancreatic volumes.[8]. |
| **Proposed pathophysiology** | Exposure to PD-1 inhibitor unmasks autoimmunity and triggers β-cell destruction. | Genetic predisposed individual exposed to an environmental trigger, leading to autoimmune β-cell destruction. |

**References**

1. Quandt, Z., A. Young, and M. Anderson, *Immune checkpoint inhibitor diabetes mellitus: a novel form of autoimmune diabetes.* Clin Exp Immunol, 2020. **200**(2): p. 131-140.

2. Segerer, H., et al., *Diabetic Ketoacidosis at Manifestation of Type 1 Diabetes in Childhood and Adolescence—Incidence and Risk Factors.* Dtsch Arztebl Int, 2021. **118**(22): p. 367-372.

3. Kyriacou, A., et al., *Is immune checkpoint inhibitor-associated diabetes the same as fulminant type 1 diabetes mellitus?* Clin Med (Lond), 2020. **20**(4): p. 417-423.

4. Sokołowska, M., A. Chobot, and P. Jarosz-Chobot, *The honeymoon phase - what we know today about the factors that can modulate the remission period in type 1 diabetes.* Pediatr Endocrinol Diabetes Metab, 2016. **22**(2): p. 66-70.

5. Wu, L., et al., *Unravelling Checkpoint Inhibitor Associated Autoimmune Diabetes: From Bench to Bedside.* Front Endocrinol (Lausanne), 2021. **12**: p. 764138.

6. de Filette, J.M.K., et al., *Immune checkpoint inhibitors and type 1 diabetes mellitus: a case report and systematic review.* Eur J Endocrinol, 2019. **181**(3): p. 363-374.

7. Erlich, H., et al., *HLA DR-DQ haplotypes and genotypes and type 1 diabetes risk: analysis of the type 1 diabetes genetics consortium families.* Diabetes, 2008. **57**(4): p. 1084-92.

8. Ross, J.J., et al., *Exocrine Pancreatic Enzymes Are a Serological Biomarker for Type 1 Diabetes Staging and Pancreas Size.* Diabetes, 2021. **70**(4): p. 944-954.
